# Resveratrol-Loaded Diacetate Fiber by Supercritical CO_2_ Fluid Assisted Impregnation

**DOI:** 10.3390/ma15165552

**Published:** 2022-08-12

**Authors:** Weiwei Zhu, Jiajie Long, Meiwu Shi

**Affiliations:** College of Textile and Clothing Engineering, Suzhou University, Suzhou 215127, China

**Keywords:** diacetate fiber, supercritical CO_2_ fluid, resveratrol, loading ability

## Abstract

(1) Background: Supercritical CO_2_ fluid (SCF-CO_2_)-assisted impregnation presents advantages on loading active drugs to polymer substrates, since it enables the realization of a drug-loaded polymer without any solvent residue. Besides, CO_2_ gas and drugs can be recycled and utilized again. Resveratrol-loaded diacetate fiber by SCF-CO_2_-assisted impregnation was done to give diacetate fiber biological activity function for enhancing its added value. (2) Methods: The effect of SCF-CO_2_ temperature, pressure and treatment time on loading ability (LA) of resveratrol onto diacetate fiber was explored by ultraviolet visible spectrophotometer. The variation of structure and property of diacetate fiber was analyzed by characterization instruments. (3) Results: LA had been increasing with SCF-CO_2_ treatment time, temperature and pressure when SCF-CO_2_ was above 70 °C, 12 MPa. The inhibiting rate of resveratrol to free radicals was affected positively by SCF-CO_2_. After resveratrol was impregnated by SCF-CO_2_ it appeared some small white granular substances on the surface of diacetate fiber. It had a good interaction between resveratrol and molecular chain of diacetate fiber. (4) Conclusions: Resveratrol was well-loaded onto the diacetate fiber by SCF-CO_2_ assisted impregnation.

## 1. Introduction

Cellulose acetate is an important derivative of cellulose with molecular formula [C_6_H_7_O_2_(OCOCH_3_)_X_(OH)_3−X_]n, where X is regarded as degree of substitution(DS) of acetyl, and n is degree of polymerization. According to the definition of fiber by ISO/FDIS 2077:2013 (E), the content of acetyl in cellulose diacetate is between 74% and 92% when X is within the range of 2.28~2.49. The diacetate fiber is an important regenerated cellulose fiber, the output of which is second only to viscose fiber. The fabric processed by diacetate fiber has bright color and appearance, is soft and comfortable to touch, lustrous and has good elasticity, no static electricity and pills. The performance is close to mulberry silk and suitable for close wear. So, it is often used to make high-end garments. The price is also lower than cupra fiber.

With the upgrading of consumption people are not only satisfied with fiber materials comfort, but also expect fiber materials to possess certain functions, such as antibacterial properties [1] and antioxidation [2]. Bioactive drugs that maintain and moisturize the skin are not only added to skin care products, but also used in clothing fabrics. For instance, vitamin C was added into textile in the form of microcapsules, thyme essential oil was applied to linen–cotton fabric using the padding method, herbal extracts and essential oils were loaded onto cotton and silk by utilizing pad-dry-cure method and micro-spraying method [3,4,5]. If bioactive drugs can be effectively impregnated into diacetate fiber by proper processing technology to give cellulose acetate a biologically active function, it will enhance the added value of diacetate fiber greatly.

Among the developed manufacturing processes, bioactive textiles mainly adopt the blending spinning method [6], sol-gelatin technology [3] and microcapsule technology [7]. The blending spinning method means the drug was added into spinning dope for melt spinning or wet spinning (≥85 °C). It can mix more bioactive drugs (2–10%), but it is not suitable for processing heat-sensitive components. Besides, the mixing of drugs can easily affect the performance of fiber. The sol-gel was prepared by an emulsion hardening technique, and microcapsules protect bioactive drugs through a coating system [8,9,10]. These involve various physical or chemical reactions and the use of organic solvents. So, the manufacturing processes are long and usually finished on the fabric by padding and coating, resulting in the worse feel of textile after washing. Specially, bioactive diacetate fiber is usually processed by electrostatic spinning technology, which uses electrostatic force to stretch fibers from a viscoelastic polymer solution. Different kinds of bioactive drug can be added into the polymer solution such as chitosan, gallic acid, vitamin A, vitamin E [11,12,13]. But the prepared diacetate fibers are nanosized and processed into mat instead of fabric. Besides, the electrostatic spinning technology is not applicable to industrial production. A more efficient method is needed to manufacture bioactive diacetate fiber.

Supercritical CO_2_ fluid (SCF-CO_2_) technology is a green processing technology. When the temperature and pressure are respectively above 31.1 °C and 7.37 MPa, CO_2_ gas can be transformed into supercritical fluid. This has a good solvability close to liquid and diffusivity close to gas and can dissolve or carry small molecule drugs into the surface and interior of substrates [14]. After the pressure drops, SCF-CO_2_ changes into gas, escapes from the substrate and is recovered, but drugs remain in the substrate, and finally give the substrate corresponding function [15,16]. The whole process is simple, green and environmentally friendly without involving the use of organic solvents. More importantly, the functional drug loading ability and its distribution onto the substrate can be regulated simply through changing temperature, pressure, and treatment time [17]. So, SCF-CO_2_ technology can be regarded as a better method to manufacture bioactive diacetate fiber compared to traditional processing methods.

In this work, diacetate fiber was utilized as substrate and resveratrol with the functions of whitening, moisturizing, anti-oxidation and anti-ultraviolet ray protection. This is regarded as a bioactive drug, which has been added into health and skin care products. Firstly, resveratrol was dissolved in SCF-CO_2_. Then, the solubilized resveratrol was carried into the diacetate fiber through SCF-CO_2_. Thus, an investigation of the impregnation of diacetate fiber with resveratrol in SCF-CO_2_ was done. The effect of processing time, temperature and pressure of SCF-CO_2_ on the loading ability of resveratrol onto diacetate fiber and the change of structure and properties of diacetate fiber treated by SCF-CO_2_ were studied carefully to provide some effective information for processing bioactive or other functional diacetate fibers with SCF-CO_2_.

## 2. Materials and Methods

### 2.1. Materials

**Figure 1:**
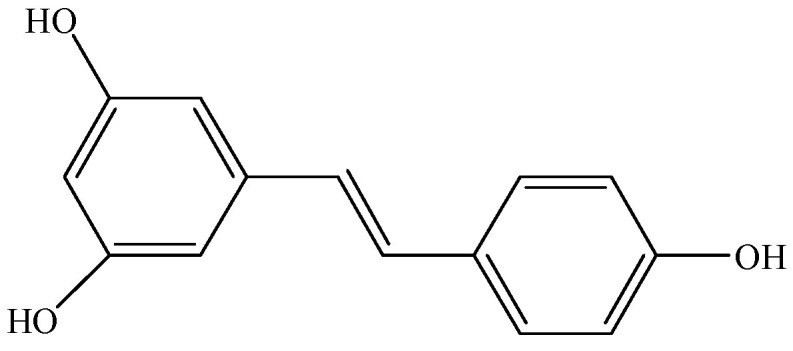
Chemical structure of the employed resveratrol drug.

The utilized resveratrol (CAS: 501-36-0) was purchased from Aladdin Co., Ltd. (Shanghai, China) in an analytical reagent grade. The chemical structure of the resveratrol is shown in Figure 1. 1,1-Diphenyl-2-picrylhydrazyl (DPPH) (CAS: 1898-66-4) was provided by Suzhou Ketong Biomedical Technology Co., Ltd. (Suzhou, China). Filament tow of diacetate fiber (2.7 dtex/f, DS = 2.45) after a full pretreatment was supplied by Nantong Cellulose Fibers Co., Ltd. (Suzhou, China). Carbon dioxide (purity ≥ 99.8 vol.%) was purchased from Chengxing Industrial Gas Supply Co., Ltd. (Suzhou, China).

### 2.2. Impregnation of Diacetate Fiber with Resveratrol in SCF-CO_2_

**Figure 2:**
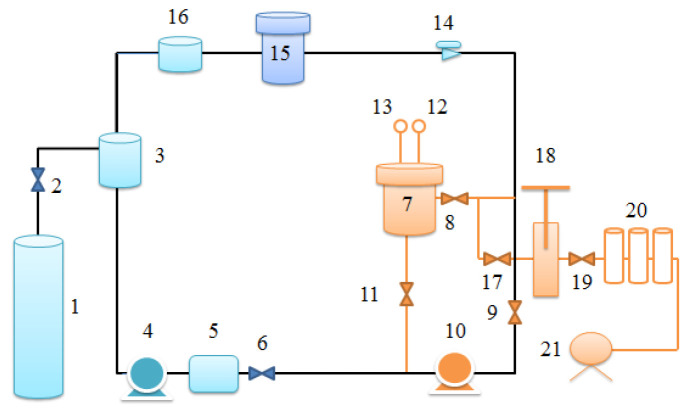
Schematic diagram of the supercritical system for diacetate fiber impregnation: (1) CO_2_ cylinder, (2, 6, 8, 9, 11, 17, 19) Valves, (3) Cooler, (4) Liquid pump, (5) Pre-heater, (7) Impregnation unit, (10) Circulation pump, (12) Pressure transducer, (13) Thermometer, (14) Micrometering valve, (15) Separator, (16) Cleaner, (18) Sampling tube, (20) Collecting units, (21) Gas flowmeter [18].

The supercritical system used for impregnation; its schematic diagrams were in the literature [18], as shown in Figure 2. Firstly, resveratrol with 5.0% by mass of diacetate fiber was utilized and was put at the bottom of the impregnation unit (7) with a free volume of 2 L, which connected with pipeline by small holes. The mass of diacetate fiber was around 4.0–5.0 g and trapped on the yarn rack, which was stood in the impregnation unit (7). There was no touching between resveratrol and diacetate fiber. After a well seal of the supercritical system, CO_2_ gas was supplied from a gas cylinder (1) and entered into the system, which began pressurizing (4) and heating up (5) until the set points that were monitored by pressure transducer (12) and thermometer (13) respectively. The fluid was circulated (10-11-7-8-9-10) at a ratio of 1:10. Namely, the circulation pump (10) was opened every 10 min to make the fluid circulate for 1 min throughout experiment, in order to dissolve resveratrol adequately. When the treatment time was up the pressure relief valve (17) was opened slowly and the pressure of impregnation unit (7) was released totally around 12 min. At the same time the heating (5) was stopped. Then, the fluid was transformed into CO_2_ gas and was recycled (15) simultaneously. Finally, resveratrol-loaded bioactive diacetate fiber was taken out from the impregnation unit (7).

### 2.3. Quantitative Evaluation of LA of Resveratrol onto Diacetate Fiber in SCF-CO_2_

The resveratrol loaded onto the diacetate fiber was extracted four times continuously by ultrasonic extraction method, and 20 mL ethanol was used as the extraction solvent at room temperature. The obtained extraction solution was transferred into a 100.0 mL volumetric flask. The extracted diacetate fiber was dried at 90.0 °C for 4.0 h and conditioned in a dryer for 24.0 h for mass measurement.

**Figure 3:**
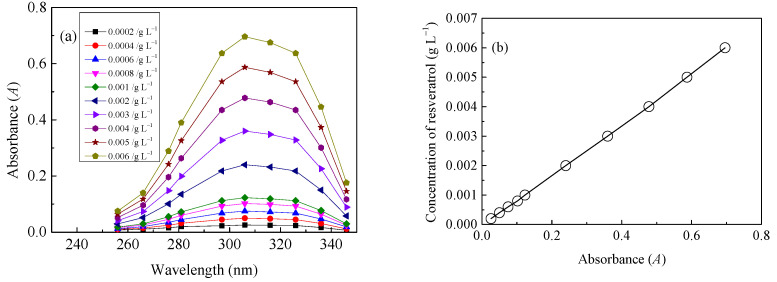
The ultraviolet spectra: (**a**) The calibration curve; (**b**) Quantitative measurement of resveratrol in a solvent of ethanol.

A construction of the calibration curve for quantitative evaluation of LA of resveratrol onto diacetate fiber was performed by preparing solutions of standard concentration. Resveratrol and ethanol were regarded as solute and solvent respectively. The absorbance of resveratrol in ethanol was measured by an ultraviolet visible spectrophotometer (TU-1810, Beijing Purkinje General Instrument Co., Ltd., Beijing, China) and the range of wavelength was 190.0 nm to 400.0 nm. Then, the calibration curve was built linearly with the characteristic absorbance against the resveratrol concentrations. The measured ultraviolet visible spectra and calibration curve were shown in Figure 3. A regression equation for quantitative measurement of the resveratrol with a characteristic absorbance at 306.0 nm was presented in Equation (1).

(1) C=0.0085×A

*C* is the concentration of resveratrol (g L^−1^). *A* represents the characteristic absorbance at 306.0 nm, corresponding to the concentration of resveratrol. Proportional peak height and a linear calibration curve in a concentration range from 2.0 × 10^−4^–6.0 × 10^−3^ g L^−1^ were shown with high linearity (r^2^ = 0.9994).

Finally, the concentration of resveratrol in the collected solvent of ethanol was known from Equation (1) according to *A* at 306 nm. Then, *LA* of resveratrol onto diacetate fiber was obtained with Equation (2), by knowing the concentration of resveratrol in the extraction solution and the mass of the utilized diacetate fiber. The relative standard deviations for determination of the *LA* were within 1.7%.

(2) $$LA=\frac{C\times V}{G}$$

where *LA* refers to the loading ability of resveratrol against utilized diacetate fiber (g g^−1^). *C* represents the concentration of resveratrol (g L^−1^). *V* is the volume of ethanol with resveratrol in the volumetric flask, and the volume of 100.0 mL was used. *G* is the mass of extracted diacetate fiber (g).

### 2.4. Free Radical Inhibiting Ratio Assessment of Resveratrol

The free radical inhibiting experiment was adopted to evaluate the functional stability of resveratrol after being treated by SCF-CO_2_. 1,1-Diphenyl-2-picrylhydrazyl (DPPH) was regarded as the free radical. Firstly, 4.0 mg of DPPH was dissolved in 80.0 mL ethanol, which was stored in a dark condition after 5.0 min of ultrasonic oscillation. A total 2.0 mL ethanol solution with DPPH and 1.0 mL pure ethanol were added into one small tube. After mixing thoroughly, we monitored the absorbance A_2_ at 517 nm on an ultraviolet visible spectrophotometer. Secondly, 20.0 mg of resveratrol was dissolved in 20.0 mL ethanol. It added v μL ethanol solution with resveratrol into the second small tube, which also had stored 2.0 mL ethanol solution with DPPH and (1000-v) μL pure ethanol. After mixing thoroughly for 30 min, it measured the absorbance A_3_ of the solution at 517 nm in the second small tube. The values of v were respectively 20, 40, 60, 80, 100. The free radical scavenging rate was evaluated according to Equation (3). The procedure was done in duplicate.

(3) $$I=\frac{A_{2}-A_{3}}{A_{2}}\times 100\%$$

where *I* was for the free radical inhibiting ratio of resveratrol (%). *A*_2_ was the characteristic absorbance of DPPH solution with no resveratrol at 517 nm. *A*_3_ was the characteristic absorbance of DPPH solution with the corresponding dosage gradient of resveratrol at 517 nm.

### 2.5. Characterization and Analysis of Diacetate Fiber

The structure and property of resveratrol-loaded diacetate fiber was characterized by a series of characterization instruments. The surface morphology was observed by using a scanning electron microscope (SEM) (Quanta-250, FEI Corporation, Brno, Czech Republic). The sample was put on the conductive adhesive for gold spray treatment and different magnifications were set. The chemical structure was analyzed by fourier transform infrared (FT-IR), which was recorded by a Nicolet 5700 spectrometer (Thermo Nicolet Corporation, Waltham, MA, USA) using the method of a potassium bromide (KBr) disc. The sample was firstly cut into powder, then mixed with KBr in an agate mortar and pressed into the tablet and dewatered under infrared light. The range was 4000~500 cm^−1^. The aggregation structure was performed by an X-ray diffraction (XRD) instrument (D/max-2550 PC, Rigaku Corporation, Tokyo, Japan). The sample was cut into powder, then made into a loose flat form and performed. Cu-Ka rays was used. The operating current was 150 mA, and operating voltage was 40 kV, 2θ was in a range of 5~60°. The thermostability was analyzed by a thermogravimetric differential thermal synthesis analyzer (TG/DTA 6300, Japan NSK Ltd., Tokyo, Japan). The sample was cut into powder and dried, and placed in the analyzer under nitrogen protection at 100.0 mL/min. The heating rate was 10 °C/min, and the temperature range was 30–700 °C. Tensile fracture strength was confirmed by a double-arm universal material testing machine (INSTRON-3365, Wistron corporation, Flower Mound, TX, USA). The pre-tension for testing was set to 0.20 cN. The tensile speed was set to 20 mm/min. The clamping distance was set to 20 mm. Each sample was tested 50 times to get average value.

## 3. Results and Discussion

### 3.1. The Effect of SCF-CO_2_ Treatment Time on LA of Resveratrol onto Diacetate Fiber

**Figure 4:**
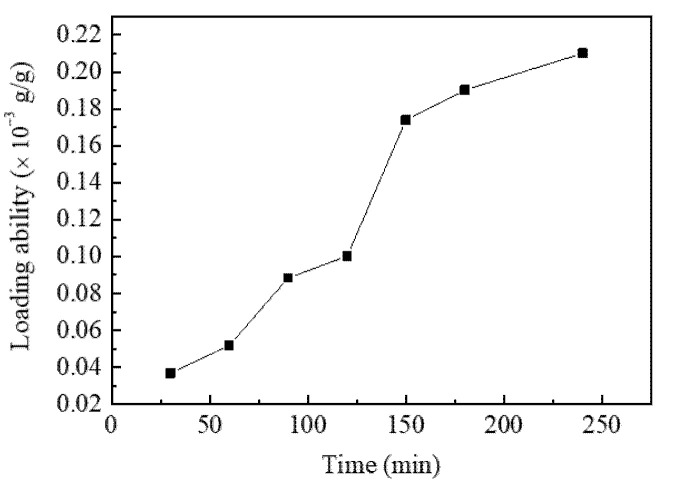
LA of resveratrol onto diacetate fiber with SCF-CO_2_ treatment time.

The resveratrol dissolved in SCF-CO_2_ was continuously penetrated into diacetate fiber under the influence of a concentration gradient between SCF-CO_2_ and diacetate fiber, so that the LA of resveratrol onto diacetate fiber will be changed with the extension of treatment time. It is necessary to explore the effect of SCF-CO_2_ treatment time on LA. The treatment times were set to 30 min, 60 min, 90 min, 120 min, 150 min, 180 min, 240 min. The temperature and pressure were respectively 80 °C, 16 MPa. The variation of LA with SCF-CO_2_ treatment time was described in Figure 4.

It can be seen that LA increased with SCF-CO_2_ treatment time. When the time was 30 min, LA was 0.0369 mg/g. When the time was 240 min LA had increased to 0.21 mg/g. The reason why the LA had been increasing over time might be that SCF-CO_2_ has a good swelling and penetrating effect on diacetate fiber. Diacetate fiber has the microstructure of a large amorphous area, a relatively small crystalline grain size and imperfect crystallization. Besides, the movement of molecular chain and chain segments is relatively free in the diacetate fiber crystal area. The non-uniform side groups of acetyl group and hydroxyl group are arranged into a relatively random manner along the macromolecular chain [19]. Finally, diacetate fiber can be swollen and penetrated easily. SCF-CO_2_ extracts functional drugs from raw materials or carries them into substrate such as spices, dyes, antibacterial agent to realize various applications. For these processes the swelling and penetrating of fluid into substrate played a great role [20,21]. Based on the above, SCF-CO_2_ entered the diacetate fiber continuously under swelling and penetrating, which improved the amount of resveratrol entering the diacetate fiber.

### 3.2. The Effect of SCF-CO_2_ Treatment Temperature and Pressure on LA of Resveratrol onto Diacetate Fiber

**Figure 5:**
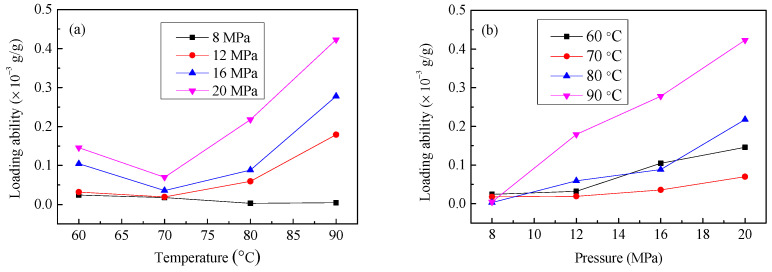
Effect of SCF-CO_2_ treatment conditions on the LA of resveratrol onto diacetate fiber: (**a**) SCF-CO_2_ treatment temperature; (**b**) SCF-CO_2_ treatment pressure.

The LA was mainly related to the solubility according to the previous experiment results, and the higher solubility resulted in higher LA of the drug onto fiber [18]. Besides, the swelling and penetrating of fluid could accelerate the passage of drug into substrate [22]. The temperature and pressure were the most critical factors for solubility or swelling [14], which determined the LA of resveratrol onto diacetate fiber. So, it was prerequisite to discuss the effect of SCF-CO_2_ treatment temperature and pressure on LA. It can be known that LA had been increasing with SCF-CO_2_ treatment time. However, the structure and property of diacetate fiber might be seriously damaged if the treatment time were too long. If diacetate fiber was to be impregnated by SCF-CO_2_ for too long a time it would become less efficient. Hence, we chose 90 min of treatment time to analyse the effect of SCF-CO_2_ treatment temperature and pressure on LA of resveratrol onto diacetate fiber. The too-high temperature and pressure of SCF-CO_2_ might easily destroy the property of diacetate fiber because of swelling and penetrating. Therefore, SCF-CO_2_ treatment temperatures were set as 60 °C, 70 °C, 80 °C, 90 °C, and the SCF-CO_2_ treatment pressures were set as 8 MPa, 12 MPa, 16 MPa, 20 MPa. The variations of LA with SCF-CO_2_ treatment temperatures and pressures are described in Figure 5.

The effect of treatment temperatures on LA under different SCF-CO_2_ pressures was depicted in Figure 5a. It shows LA decreased a little with increasing temperature when the pressure was 8 MPa. LA decreased until 70 °C and increased with increasing temperature above 70 °C when the pressure was respectively 12 MPa, 16 MPa, 20 MPa. It can be seen that LA decreased at the beginning and increased subsequently with increasing SCF-CO_2_ treatment temperatures, except when the pressure was 8 MPa. One reason is that the variation of LA was positively related to solubility, a higher solubility resulted in a higher LA. The solubility was closely related to SCF-CO_2_ temperature, which decreased with increasing SCF-CO_2_ temperature under transition pressure [23]. Transition pressure was usually 10–12 MPa. So, the decreasing solubility with increasing temperature caused a decreasing LA when the pressure was 8 MPa. When the pressure was above 12 MPa, LA still decreased with SCF-CO_2_ temperature going up from 60 °C to 70 °C, but increased from 70 °C to 90 °C. The reason could be that the fluid density reduced with increasing temperature in a small scale at a constant SCF-CO_2_ pressure. It resulted that the swelling and penetrating of SCF-CO_2_ to diacetate fiber got worse, which affected the entry of resveratrol into cellulose acetate. However, when the temperature was above 70 °C the swelling of SCF-CO_2_ was greatly enhanced. The solubility of resveratrol also increased with increasing temperature. Under the combined effect of the two factors, LA was improved when SCF-CO_2_ temperature raised up from 70 °C to 90 °C.

The effect of SCF-CO_2_ treatment pressure on the LA was described in Figure 5b. It can be seen that LA increased with increasing pressure under different SCF-CO_2_ temperatures. One reason was that density of CO_2_ was enhanced with increasing pressure so that the solubility of resveratrol in SCF-CO_2_ was improved. A higher solubility resulted in a higher LA. The other reason was that the swelling and penetrating of SCF-CO_2_ into diacetate fiber caused it to become enlarged with increasing fluid density. It resulted that more resveratrol entered into the diacetate fiber. Finally, LA was increasing with SCF-CO_2_ pressure.

### 3.3. Free Radical Inhibiting Ratio of Resveratrol to DPPH after Being Treated by SCF-CO_2_

**Figure 6:**
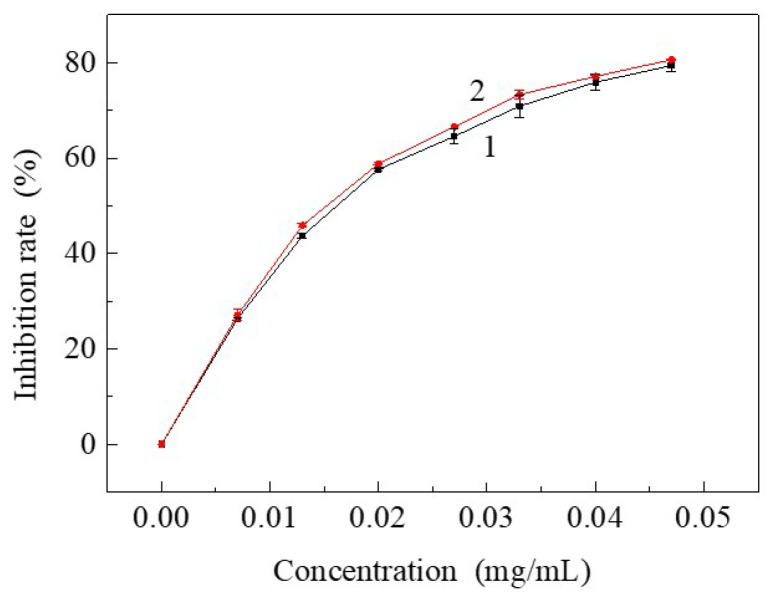
The inhibiting rate of resveratrol to DPPH: (1) Untreated resveratrol; (2) Resveratrol treated by SCF-CO_2_ at 80 °C, 20 MPa, 90 min.

If resveratrol was soaked in SCF-CO_2_ it might affect the activity of resveratrol. So, it was necessary to explore the bioactive functions of resveratrol after being treated by SCF-CO_2_, which was evaluated by inhibiting rate of resveratrol to DPPH. It showed that the horizontal coordinate was the concentration of resveratrol added to the DPPH and the vertical coordinate was the inhibiting rate of resveratrol to DPPH in Figure 6. It can be noticed that the inhibiting rate of resveratrol treated by SCF-CO_2_ to DPPH was slightly higher than untreated sample. This suggests that bioactive function of resveratrol was not affected after being treated by SCF-CO_2_. The higher inhibiting rate may be due to that SCF-CO_2_ played a role in purification of resveratrol. Although the utilized resveratrol was analytically pure there still existed several impurities after synthesis with different kinds of organic solvents, which could be dissolved and taken away by SCF-CO_2_ with a good solubility to non- or low-polar compounds. Finally, the improved purification of resveratrol resulted in the higher inhibiting rate of resveratrol to DPPH.

### 3.4. Structure and Property of Resveratrol-Loaded Diacetate Fiber

#### 3.4.1. Distribution Pattern of Resveratrol on the Surface of Diacetate Fiber

**Figure 7:**
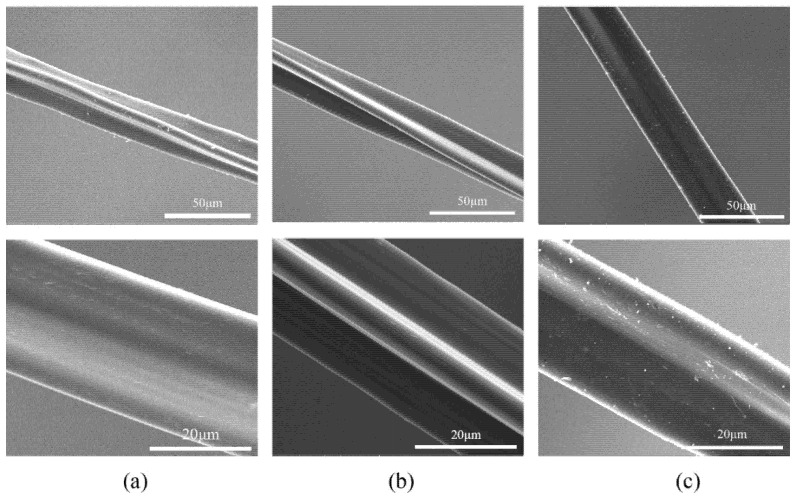
Distribution pattern of resveratrol on the surface of diacetate fiber: (**a**) Untreated diacetate fiber; (**b**) Diacetate fiber treated by SCF-CO_2_ at 80 °C, 20 MPa, 90 min; (**c**) Impregnation of diacetate fiber with resveratrol in SCF-CO_2_ at 80 °C, 20 MPa, 90 min.

The distribution pattern of resveratrol on the surface of diacetate fiber was observed by SEM, which were magnified by 1000 times and 3000 times respectively, as shown in Figure 7. It can be seen that the diacetate fiber was a triangular-shaped fiber with deep grooves and convex edges. The morphology had no distinct change, but the surface smoothness was significantly improved after SCF-CO_2_ treatment. Because CO_2_ molecules were non-polar molecules, SCF-CO_2_ had a good dissolving effect on oils and fats according to the principle of similar compatibility [24]. With the circulation of SCF-CO_2,_ the oil particles on the surface of diacetate fiber were dissolved and separated from the surface of the diacetate fiber. The impurities were also taken away. Finally, the smoothness of diacetate fiber was improved. However, after resveratrol was loaded onto diacetate fiber there appeared some small white granular substances on the surface of diacetate fiber, which was resveratrol. This suggests that dissolved resveratrol entered into diacetate fiber in SCF-CO_2_, but after decompression resveratrol was precipitated in the form of small white granules.

#### 3.4.2. Chemical Structure of Resveratrol-Loaded Diacetate Fiber

**Figure 8:**
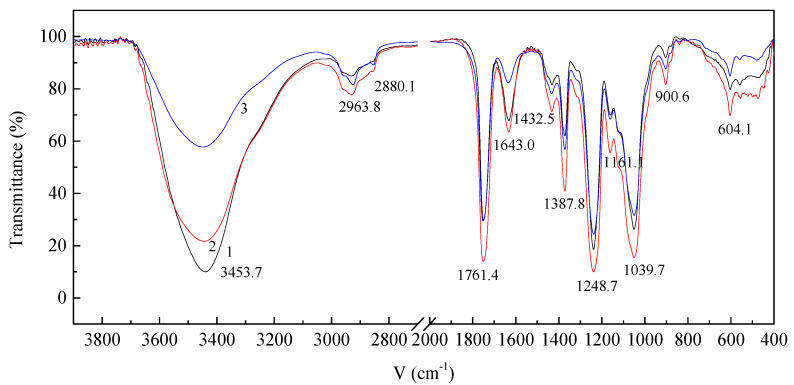
FT-IR spectra of diacetate fiber: (1) Untreated diacetate fiber; (2) Diacetate fiber treated by SCF-CO_2_ at 80 °C, 20 MPa, 90 min; (3) Impregnation of diacetate fiber with resveratrol in SCF-CO_2_ at 80 °C, 20 MPa, 90 min.

The variation in chemical structure of diacetate fiber loaded with resveratrol was investigated by FT-IR, as shown in Figure 8. The peaks is assigned to O-H stretching vibration and C=O stretching vibration respectively in the range of 3100 cm^−1^–3700 cm^−1^ and 1800 cm^−1^–1700 cm^−1^. The asymmetric and symmetric stretching vibration of C-H bonds of -CH_3_ groups, -CH_2_- groups are respectively around 2963.8 cm^−1^, 2880.1 cm^−1^. The asymmetric and symmetric stretching vibration of C=O bonds in ester groups are about 1761.4 cm^−1^, 1643.0 cm^−1^. The peak at 1387.8 cm^−1^ is the bending vibration of C-H bonds in an -O(C=O)-CH_3_ group. The peak about 1248.7 cm^−1^ is the stretching vibration of C=O bonds of acetyl group. The peaks at 1161.1 cm^−1^, 1039.7 cm^−1^, 900.6 cm^−1^ are respectively C-O-C asymmetric bridge stretching of glucose ring bridge groups, C-O-C stretching of the pyranoid ring, wagging vibration of C-H bonds [25,26].

It can be noticed from Figure 8 that the intensity of O-H stretching vibration of diacetate fiber treated by SCF-CO_2_ decreased. After impregnation with resveratrol the intensity of O-H stretching vibration of diacetate fiber was weakened further. The intensity of symmetric stretching vibration of C=O bonds in ester groups at 1643.0 cm^−1^ also decreased. The results of FTIR show that the original hydrogen bonds between the molecular chains of diacetate fiber, treated only by SCF-CO_2,_ were partially broken. The impregnation of resveratrol further destroyed the hydrogen bond. This suggests that SCF-CO_2_ weaken the interaction between molecular chains of diacetate fiber and had a plasticizing effect on diacetate fiber. When resveratrol was impregnated into diacetate fiber, there occurred the hydrogen bond interaction between resveratrol and diacetate fiber so that the intensity of O-H stretching vibrations decreased further. In addition, the hydrogen bond interaction between C=O bonds in ester groups of diacetate fiber and the O-H bonds of resveratrol resulted in the decreased intensity of stretching vibration of C=O bonds.

#### 3.4.3. Aggregation Structure of Resveratrol-Loaded Diacetate Fiber

**Figure 9:**
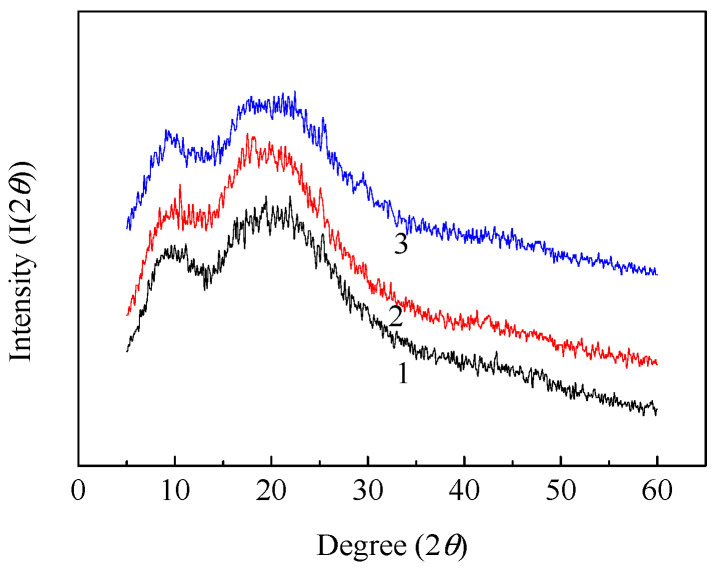
XRD patterns of diacetate fiber:(1) Untreated diacetate fiber; (2) Diacetate fiber treated by SCF-CO_2_ at 80 °C, 20 MPa, 90 min; (3) Impregnation of diacetate fiber with resveratrol in SCF-CO_2_ at 80 °C, 20 MPa, 90 min.

The variation of aggregation structure of resveratrol-loaded diacetate fiber was investigated by XRD, as shown in Figure 9. It can be observed that there were two apparent diffraction peaks at 2θ = 9.639° (210), 2θ = 19.361° (021). There existed a large proportion of non-crystal area in diacetate fiber, and the diffraction peaks were wide and not sharp. This indicated that the crystal phase was incomplete. It had been calculated that the crystallinity of untreated diacetate fiber, diacetate fiber treated by SCF-CO_2_, resveratrol-loaded diacetate fiber was respectively 29.93%, 23.32%, 18.81%. From the spectra a decreasing intensity of diffraction peak was observed at 2θ = 9.639° when diacetate fiber was treated only by SCF-CO_2_. The intensity also descended at 2θ = 19.361° after resveratrol was impregnated into diacetate fiber by SCF-CO_2_. This suggests that it had a plasticizing effect on diacetate fiber in SCF-CO_2_, which was further strengthened when resveratrol was impregnated into diacetate fiber by SCF-CO_2_. Finally, it resulted in the decreasing intensity of the diffraction peak and the ordered structure of molecular chain of diacetate fiber partially transformed into a disordered structure. The fundamental reason for this was that the entry of CO_2_ small molecules and resveratrol weakened the interaction between diacetate fiber molecular chains, which was also verified by FT-IR.

#### 3.4.4. Thermal Degradation of Resveratrol-Loaded Diacetate Fiber

**Figure 10:**
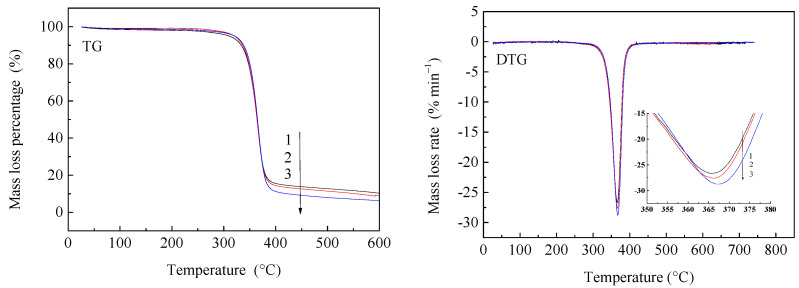
TG-DTG curve of diacetate fiber (1) Untreated diacetate fiber; (2) Diacetate fiber treated by SCF-CO_2_ at 80 °C, 20 MPa, 90 min; (3) Impregnation of diacetate fiber with resveratrol in SCF-CO_2_ at 80 °C, 20 MPa, 90 min.

The variation of thermal degradation of untreated diacetate fiber, treated diacetate fiber, and resveratrol-loaded diacetate fiber is shown in Figure 10. It can be seen that the differences of initial decomposition temperature were small for the three samples from the TG curve. However, the thermal weight loss rates were changing, which were respectively 89.72%, 90.48%, 92.89% through diagraph analysis. Meanwhile the maximum decomposition rate for the three samples were respectively 26.66%/min, 27.61%/min, 28.74%/min, and the corresponding temperatures were 365.4 °C, 366.1 °C, 367.1 °C from DTG curve. This suggests that the thermal weight loss rate and the maximum decomposition rate for untreated diacetate fiber were lower than two other samples treated by SCF-CO_2_, which were the highest for resveratrol-loaded diacetate fiber. The results indicated that the thermostability of diacetate fiber treated only by SCF-CO_2_ decreased slightly. When resveratrol was impregnated into diacetate fiber by SCF-CO_2_ the thermostability of diacetate fiber was reduced further. From the results of FT-IR spectra and XRD patterns it can be known that the intensity of O-H stretching vibration was weakened between molecular chains of diacetate fiber and a decreasing crystallinity was occurred when resveratrol was impregnated into diacetate fiber by SCF-CO_2_. This indicated that the variation of microstructure of diacetate fiber resulted in a slight decrease in thermal stability.

#### 3.4.5. Tensile Breaking Strength of Resveratrol-Loaded Diacetate Fiber

The tensile breaking strength of diacetate fiber is illustrated in Table 1, and the variation rate means the drop percentage in breaking strength of treated diacetate fiber against the untreated sample. It was observed that tensile breaking strength of diacetate fiber treated by SCF-CO_2_ decreased slightly, which declined further after diacetate fiber was impregnated with resveratrol. The reason was that the variation of microstructure of diacetate fiber after being treated. FTIR and XRD showed some hydrogen bonds were destroyed and the ratio of crystalline regions of diacetate fiber after treatment was scaled down. This meant that the interaction between diacetate fiber molecular chains was weakened, which caused a decrease in the tensile breaking strength of diacetate fibers.

## 4. Conclusions

High-efficiency, green, environmentally friendly SCF-CO_2_ was utilized to prepare bioactive diacetate fiber. The processing condition for resveratrol-loaded diacetate fiber and its variation of structure and property were investigated. An increased SCF-CO_2_ treatment time, temperature, and pressure resulted in a raised LA. Anti-free radical property of resveratrol after being treated by SCF-CO_2_ was improved. When the SCF-CO_2_ was 80 °C, 20 MPa, 90 min resveratrol could be well impregnated into diacetate fiber. However, it had a negative effect on microstructure and macro performance of diacetate fiber. This suggests that SCF-CO_2_ can be regarded as an effective method to prepare bioactive or other functional diacetate fiber, but that the suitable processing conditions need to be explored further in the future.

## Figures and Tables

**Table 1:** Tensile breaking strength of diacetate fiber samples.

Sample	Tensile Breaking Strength (cN)	Variation Rate (%)
(1)	3.20	
(2)	2.97	−7.19
(3)	2.07	−35.31

Remark: (1) Untreated diacetate fiber; (2) Diacetate fiber treated by SCF-CO_2_ at 80 °C, 20 MPa, 90 min; (3) Impregnation of diacetate fiber with resveratrol in SCF-CO_2_ at 80 °C, 20 MPa, 90 min.
